# Determinants of prescribing behaviour of antibacterial drugs in Europe and use of appropriate nomenclature in the literature

**DOI:** 10.1007/s00210-025-04511-2

**Published:** 2025-08-13

**Authors:** Lilly Josephine Bindel, Roland Seifert

**Affiliations:** Institute of Pharmacology, Hannover Medical School, 30625 Hannover, Germany

**Keywords:** Antimicrobial consumption, Antibiotic, Antibacterial drug, AMR, AMC, Antibiotic prescription, Europe, Surveillance, Antibiotic stewardship, Rational prescribing behaviour, Irrational prescribing behaviour, One health, EU, AWaRe, Health system

## Abstract

Antibacterial drugs are used in the treatment of bacterial infections. Increasing bacterial resistance rates are threatening their efficacy and place a huge burden on public health. There is a strong link between consumption and irrational prescribing behaviour versus the development of bacterial resistance. This review summarises important aspects of European prescribing behaviour for antibacterial drugs in primary care. Attributes of rational and irrational prescribing behaviour are explained. The most important determinants are investigated and discussed. An update on the use of appropriate nomenclature is also provided. Based on the findings, proposals for action are presented. Unlike other studies that focus on single determinants or prescribing behaviour on an individual level, this review considers prescribing behaviour at a systemic level. A PubMed search was conducted for search terms of prescribing behaviour. Inclusion criteria were literature published in the last five years (starting from 2020), antibacterial drugs, prescribing behaviour and related factors, Europe or European countries, and primary care. Studies solely referring to the hospital sector were excluded. This is followed by an update to the nomenclature investigation, referring to a previous analysis by Seifert and Schirmer (Naunyn-Schmiedebergs Arch Pharmacol 394:2153–2166 (2021)). Prescribing behaviour is shaped by multiple interrelated systemic and individual-level factors. Systemic factors relate to the structure of the healthcare system, whereas individual determinants refer to the single treatment decisions. In Europe, there is a strong North–South shift, with more rational prescribing in the North and problematic behaviour in the South. No significant improvements in decreasing use or appropriate drug choice have been observed in recent years, and the impact of the pandemic on prescribing practices is negative. The use of appropriate nomenclature in the literature did not improve, with a dominance of problematic terms. Policy measures are considered the most effective way of changing prescribing behaviour. Systemic changes are required before individual prescribing behaviour can improve. Conducted interventions had a positive effect in most cases but varied in their effect. Initial actions include restricting the use of antibacterial drugs to specific indications, making financial changes to encourage the use of first-line drugs, and improving infrastructure to enable targeted treatment through diagnostics. However, regulatory restrictions must be accompanied by guidance and stewardship programmes to improve adherence. In general, reforming the nomenclature in the literature is necessary to improve communication and prevent misunderstandings. Rational prescribing behaviour is a requirement for achieving a decrease in bacterial resistance.

**Supplementary Information:**

The online version contains supplementary material available at 10.1007/s00210-025-04511-2.

## Introduction

**Fig. 1:**
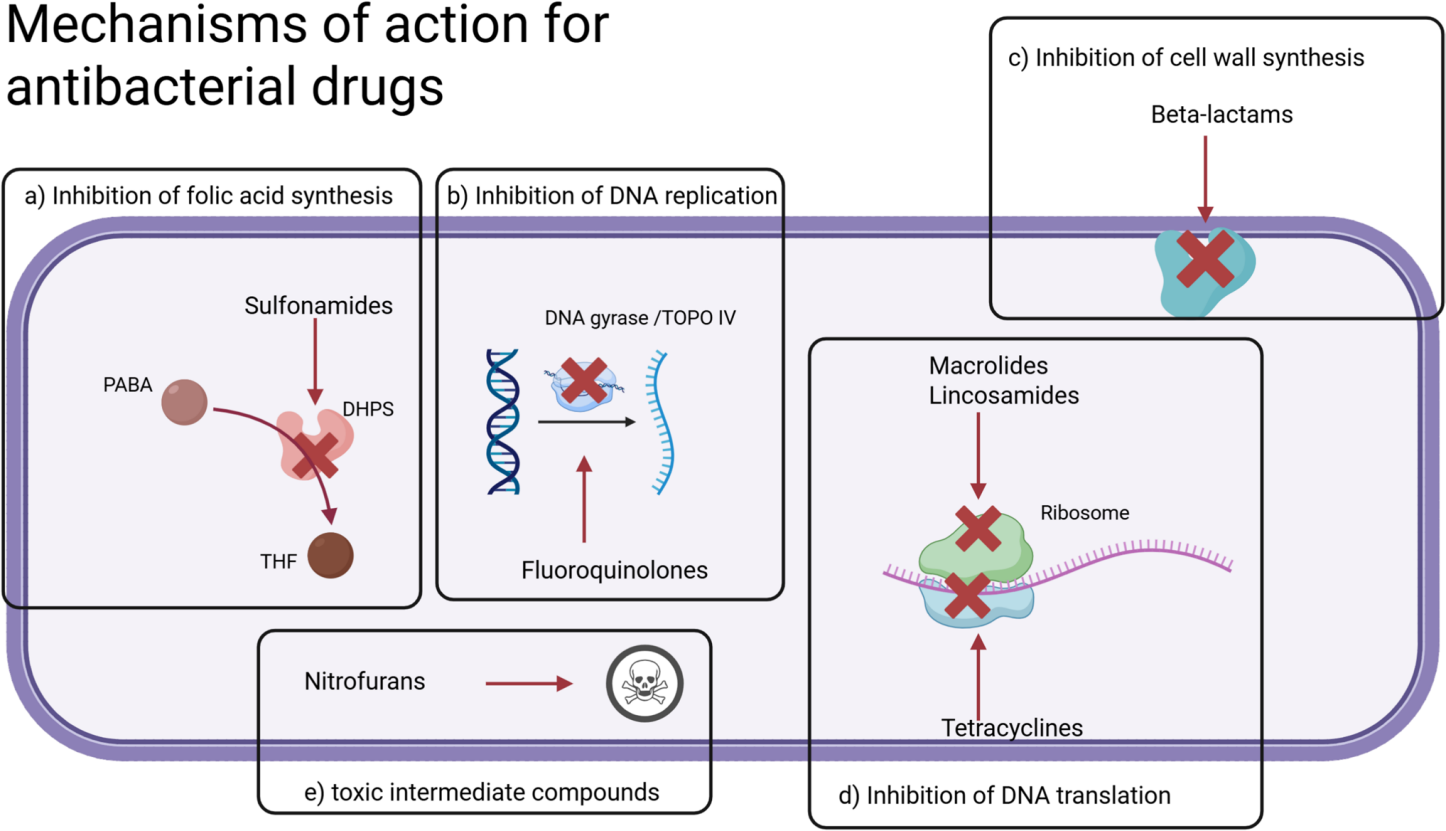
Graphical illustration of the main mechanisms of action of commonly used antibacterial drug classes. The targeted metabolic processes in bacterial cells are depicted. Sulfonamides act by inhibiting dihydropteroate synthase (DHPS), the first enzyme in the reaction that transforms para-aminobenzoic acid (PABA) into tetrahydrofolic acid (THF). This consequently inhibits folic acid synthesis (**a**) (Hilal-Dandan and Brunton, 2016). Fluoroquinolones inhibit DNA gyrase and topoisomerase IV, thereby preventing DNA replication (**b**). Beta-lactams inhibit multiple enzymes responsible for cell wall synthesis (**c**). Tetracyclines inhibit the 30S unit of the ribosome, thereby inhibiting DNA translation (**d**), as do macrolides and lincosamides, which inhibit the 50S unit. Nitrofurans are converted into toxic intermediate compounds (**e**) that damage the cell. The following references were used as a template: Caioni et al. 2024; Sanseverino et al. 2018. The graphic was designed in BioRender (https://BioRender.com/48cx0t5)

**Fig. 2:**
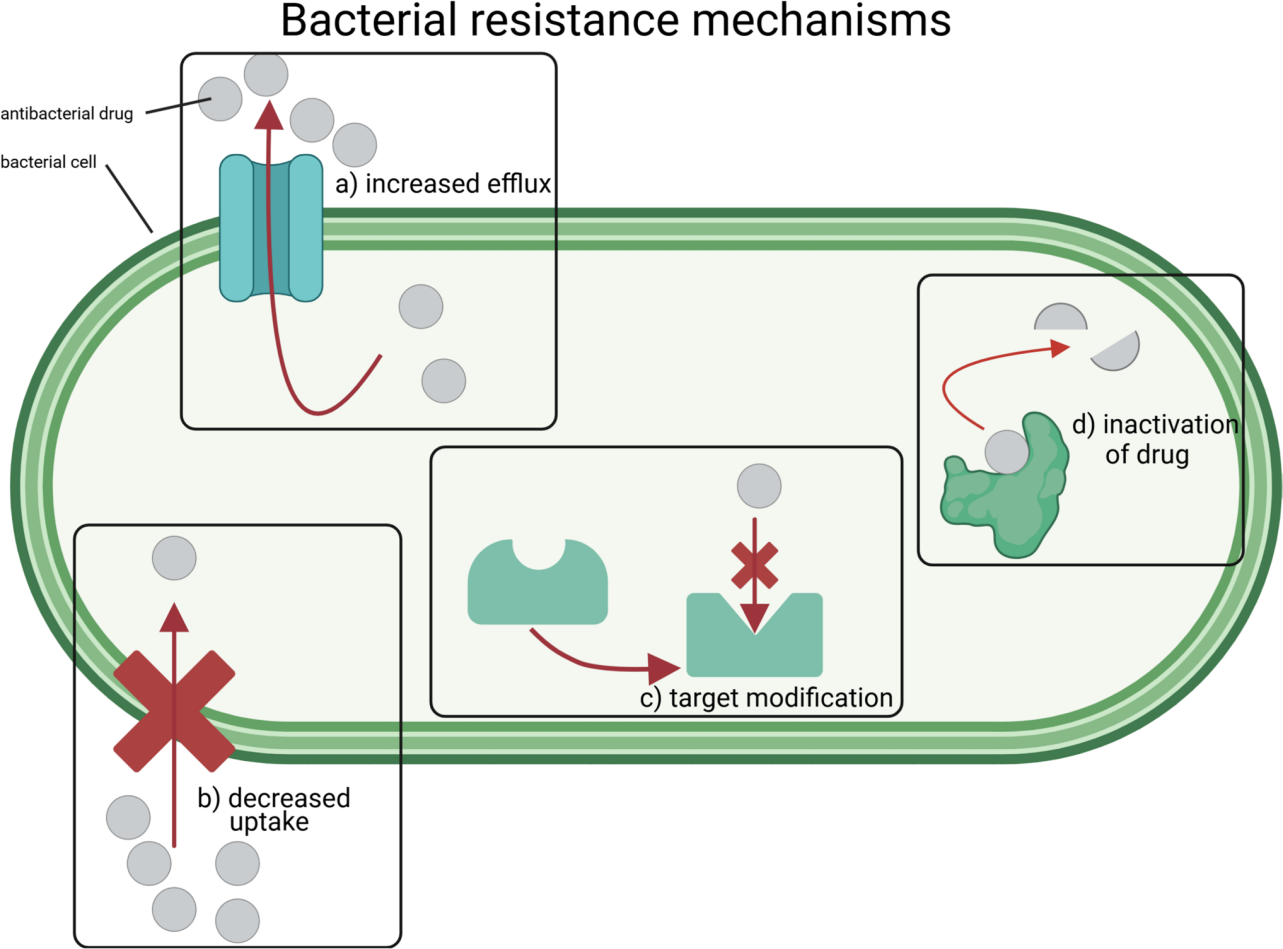
Graphical illustration about the 4 main mechanisms of bacterial resistance against antibacterial drugs. They include increased efflux mechanisms (**a**), decreased uptake of the drug (**b**), target modification (**c**) and inactivation of the drug (**d**). The following references were used as a template: Mutuku et al. 2022; Alvarez-Martinez et al. 2020; Darby et al. 2023. The graphic was designed in BioRender (https://BioRender.com/rcdnh17)

Antibacterial drugs are used in medicine for the treatment of bacterial infections (Werth 2025). Since their discovery in the twentieth century, they have contributed to the progress of modern medicine (Hutchings et al. 2019). Their effect relies on mechanisms that target metabolic processes in bacterial cells, such as cell wall synthesis, DNA replication, or translation (Seifert 2018; Sarkar et al. 2017; Santos and Lamers 2020; Barrenechea et al. 2021) (Fig. 1). However, bacteria developed strategies to overcome the bacteriostatic or bactericidal effects of antibacterial drugs, either through natural resistance or acquired mechanisms (Podolsky 2018; Halawa et al. 2024) (Fig. 2). Worryingly, bacterial resistance to antibacterial drugs has become a serious problem in recent decades, with increasing rates of resistance (Ventola 2015; Salam et al. 2023; GBD 2021 Antimicrobial Resistance Collaborators 2024; Ventura-Gabarró et al. 2023; ECDC 2024).

Rational prescribing of drugs is considered a fundamental principle of good clinical practice (Maxwell 2016). Yet, the rise in bacterial resistance highlights persistent issues in the prescribing of antibacterial drugs. There is a strong link between the development of bacterial resistance and both the volume and the inappropriate use of these drugs (ECDC 2024; Abejew et al. 2024; Bindel and Seifert 2025b, c). Increasing bacterial resistance comes with severe consequences, such as increasing morbidity and mortality, substantial healthcare costs and an increasing burden for public health in general (Antimicrobial Resistance Collaborators 2022). Therefore, rational prescribing is considered essential for solving problems related to antibacterial resistance.

Previous research by our group has identified several important drivers of antibacterial consumption and prescribing behaviour, such as economic considerations (Bindel and Seifert 2024a, b), cultural attitudes, and regional differences (Bindel and Seifert 2025b, c). In a previous study, Schirmer and Seifert (2021) showed that use of inappropriate nomenclature is an important factor for irrational prescription of antibacterial drugs. This aspect is assessed again to reflect changes and improvements since the earlier study.

The review aims to summarise and investigate current patterns of antibacterial drug use and prescribing behaviour in Europe, with a particular focus on identifying and analysing the key determinants that influence these practices. Therefore, a PubMed search was conducted to collect key literature on these topics. Relevant drivers and influencing factors were identified and evaluated in the context of recent developments. Based on this analysis, proposals for action were developed to effectively address problematic use and improve prescribing.

## Materials and methods

### Investigated subject and PubMed search

The focus of this review was set on prescribing behaviour and its determinants for antibacterial drugs in Europe. Therefore, a literature search was performed in ‘PubMed’ for the following search terms: (‘rational prescribing’) AND (‘antibiotics’ OR ‘antibacterial drugs’); (‘irrational prescribing’) AND (‘antibiotics’ OR ‘antibacterial drugs’); (‘prescribing behaviour’) AND (‘antibiotics’ OR ‘antibacterial drugs’); (‘appropriate use’) AND (‘antibiotics’ OR ‘antibacterial drugs’); (‘consumption’) AND (‘antibiotics’ OR ‘antibacterial drugs’) AND (‘Europe’); (‘cultural attitudes’) AND (‘consumption’) AND (‘antibiotics’ OR ‘antibacterial drugs’).

Included were publications from the last 5 years (starting from 2020) and regarding Europe or European countries. Since the focus was set on the outpatient sector, studies that only regarded the hospital sector were excluded.

### Update of the findings of Seifert and Schirmer (2021)

To investigate another aspect of rational prescribing, the use of an appropriate nomenclature, we updated the findings of the analyses conducted by Seifert and Schirmer (2021). The original analysis compared the use of appropriate versus problematic nomenclature by investigating the frequency of used terms in scientific articles indexed in PubMed.

In the update, the findings provided in their supplementary material were extended for the next 4 years (2020–2024). This included the number of citable items in PubMed, the absolute number of citable items for the respective search term, and the normalised count. Further details about the methodology and search terms can be found in the supplement.

### Analysis of key literature and implementation of proposals for action

Based on the findings of the PubMed search, literature for important aspects on prescribing behaviour of antibacterial drugs was collected (Tables 1, 2, 3, 4 and 5). The key results about the review topic were discussed and supplemented by a general literature search to provide further verification and evidence of important aspects. Finally, the results were interpreted and basic proposals for action to improve prescribing behaviour were provided.

**Table 1:** Key findings from the literature review on the prescribing behaviour of antibacterial drugs. For each reference, information on the subject, region and the most important findings and their value, as well as the limitations, is provided

Subject	Region	Take-home messages	Value of the findings	Limitations of the study	Reference
Antibacterial consumption	Europe	Strong geographical differences in consumption volume and trend, North-West and South-East shift; poor progress to national targets in many countries	Trend analysis of past years and forecast until 2030; highlights particularly problematic regions	Varying forecast reliability; general assessment	Bindel and Seifert 2025c
Antibacterial consumption and prescribing practice	Iceland	Overall decrease in consumption; strong adherence to guideline recommendations; consistent with comparably well prescribing behaviour of Nordic countries	Success of national programme with guidance and feedback of the treatment of common infections; ability to reduce consumption and inappropriate prescribing	Short observation period, only paediatric setting considered	Gunnlaugsdottir et al. 2021
Antibacterial consumption and targets for stewardship programmes	France	High consumption in the outpatient sector, despite stewardship programmes; higher prescriptions are associated with patients being female, of advanced age, having comorbidities; high prescriptions of general practitioners, dentists and paediatricians; slight decrease in consumption over time	Identification of prescribing indicators and target groups; focus on main prescribers to encourage appropriate and low use	Impact of the COVID pandemic might be an alternative explanation for the decrease in consumption; data limitations	Bara et al. 2022
Determinants of antibacterial prescribing in general practitioners	Italy	Direct factors influencing general practitioners prescribing: socio-demographics, knowledge, attitudes, patent-related factors, pharma influence, healthcare system and infrastructure	Identification of intrinsic and extrinsic Factors on clinical practice; potential targets for interventions	No investigation of the reason for the patients visit resulting in a prescription is available	Kurotschka et al. 2020
District-level differences in prescribing	Germany	Regional variations, not fully explained by socioeconomic and health care density differences; low-prescribing regions reported supporting contextual factors and resources; high-prescribing regions have poor coordination between services, lack of knowledge; variations in patient expectations	Interventions promising that target professional collaboration, infrastructure and guidelines; consideration of region-specific attitudes	Subjective perceptions of participants because of self-reports	Schülz et al. 2024
Drivers of overprescribing in general practitioners	global	Main factors are attitudes of practitioners like fear or anxiety, external factors like time and financial pressure, patient pressure, lack of education, non-adherence to guidelines; complex interrelated factors; multifaceted approach needed	Identification of determinants which can be addressed by stewardship interventions; suggestions to lower consumption; can be an addition to existing programmes; need for regular training, public awareness and knowledge; improvements of external factors	Reliance on strengths and methodology of the analysed studies; restrictions by methodological settings like inclusion and exclusion criteria	Rose et al. 2021
Factors associated with irrational prescribing	Scotland	Overprescribing for respiratory tract infections; variations of interpretations of diagnostics among physicians, some of them being linked to inappropriate prescribing	Distinguishing between automatic and analytical prescribing; physicians need more guidance for interpretation of symptoms related to antibacterial drug use; interventions shall target differing interpretation and both modes of thinking	Number of participants; restricted consideration of other determinants	McClearly et al. 2021
Prescribing behaviour	Europe	North–South and East–West shift in European prescribing, with high bacterial resistance in the South-East; poor progress in problematic regions; regional differences; tailored interventions necessary	Focus on prescribing quality by using the AWaRe framework; projections to guide policy measurements	Limitation of the AWaRe classification; varying forecast reliability	Bindel and Seifert 2025d
Prescribing behaviour	Germany	Stronger influence of costs than bacterial resistance on consumption; irrational or semi-rational prescribing of analysed drugs	Necessity to consider economic influences in stewardship programmes	Limited to Germany; restricted generalisation due to the analysis of single antibacterial drugs	Bindel and Seifert 2024b
Prescribing behaviour in children	Italy	High use of antibacterials in acute infections; unnecessary use causes costs and rising bacterial resistance	Provides recommendations for rational prescribing; emphasises the importance of rational prescribing and consequences of irrational use	Data is limited to Italy and children prescribing; observational design	Aricò et al. 2023
Prescribing of beta-lactams	Spain	High reporting of penicillin allergy although a minority has an allergy; reassessment of allergy improves rational prescribing, lowers bacterial resistance and costs	Investigation how delabeling of allergic patients and safe prescription of beta-lactams can be achieved; provides guidance	Regained possibility of appropriate prescribing does not necessarily ensure rational prescribing; no assessment in real-world setting	Arriazu et al. 2024

**Table 2:** Key findings from the literature review regarding the knowledge of patients and professionals about the rational use of antibacterial drugs and bacterial resistance. For each reference, information is provided on the subject, region, the most important findings and their value, and the limitations

Subject	Region	Take-home messages	Value of the findings	Limitations of the study	Reference
Knowledge in healthcare workers	France	Lack of knowledge concerning antibacterial prescribing and bacterial resistance; theoretical knowledge depends on the curriculum; after training improvement of rational use and practical skills	Comprehensive and frequent training in students improves/is important for rational prescribing; has to be implemented in the curriculum	Self-reporting about knowledge and skills, no objective testing; limited generalisation due to differing education of healthcare professionals between countries	Slekovec et al. 2023
Knowledge in healthcare workers	Germany	Medical students don't feel sufficiently trained to use antibacterials wisely; interested students were not able to treat rationally due to gaps in knowledge and practical skills	Teaching formations shall emphasise clinical applications, communication and feedback from instructors	Low response rate in online surveys; potential selection bias; findings restricted to German education	Wiese-Posselt et al. 2023
Knowledge in healthcare workers	Serbia	Knowledge about antibacterials available; recoding of misconceptions	Strengthened education necessary to improve understanding, perceptions, problematic use	Self-reports by questionary; findings restricted to Serbian education	Horvat et al. 2020
Knowledge in patients	Italy	Awareness is associated with good level of education, healthcare occupation and low anxiety; no strong awareness of bacterial resistance; good doctor-patient communication necessary	Highlights need to improve knowledge on antibacterials, bacterial resistance and treatment of infections	Sample is not representative of general population; no objective method for anxiety level; health literacy skills were not evaluated	Zaniboni et al. 2021
Role of patient education	global	Education increases knowledge and awareness about antibacterial drugs; can reduce demand; no effect of learning format	Demonstrates need and strategy for public awareness campaigns; highlights possibilities and positive effects; proving practical recommendations	No specification of optimal strategy	Hunter and Owen 2025

**Table 3:** Key findings from the literature regarding regulatory measurements, policy interventions and stewardship programmes related to antibacterial drugs. For each reference, information on the subject, region, the most important findings and their value and the limitations is provided

Subject	Region	Take-home messages	Value of the findings	Limitations of the study	Reference
Antibacterial stewardship and consumption during COVID	Global	High consumption of antibacterials during the COVID pandemic; stewardship improves rational and lower prescribing	Identification of key measures to improve stewardship; provides overview of promising measurements and compares their effect	Lack of evaluation of implementation; need for standardised assessment of effect	Elshenawy et al. 2023
Appropriate use of last-resort antibacterial drugs	Global	Access to effective antibacterials for multidrug-resistant pathogens is important; should be coupled with diagnostics, trained professionals and functional policy programmes; challenge to balance access and excessive use	Ensuring responsible use of last-resort antibacterials; guiding responsible use by highlighting important interventions	No exploration in real-world setting; theoretical principles	Patel et al. 2024
Assessment of the strategy for ‘delayed antibacterial prescription’	United kingdom	Delayed prescriptions might be an acceptable approach to reduce antibacterial consumption, particularly for key groups and infections; improved knowledge important for patient acceptance	Comparison between normal prescription and patient advise to delay initiating antibacterial	Hypothetical design; no comparison with other stewardship approaches	Morrell et al. 2021
Broader concepts of sustainable stewardship	Global	Suggesting a context-adapted stewardship that emphasises the right antibacterial at the right time for the right duration at an affordable price; adopting strategies from the management of other diseases; programmes not effectively implemented in practice	Improving stewardship from a resource intensive and cost-containment to a broader concept of sustainable access; identification of intervention areas to support appropriate use, optimal access and maximisation of resources; developing a comprehensive stewardship model	Theoretical model; not implemented in clinical practice	Cohn et al. 2024
Harmonisation of product information	European Union	Regulatory procedures led to restrictions in antibacterial use, updates to dosing, contraindications and warnings; promotes rational use; harmonisation ensures consistent and rational use across member states; frequent updates are important	Regulatory frameworks are important in product information updates to improve rational prescribing; highlights need for regulatory harmonisation to improve antibacterial use and lower bacterial resistance	Does not investigate the direct impact on prescribing behaviour	Opalska et al. 2020
Impact of guidelines on prescribing behaviour	Croatia	Improvement of physicians’ knowledge; often inappropriate use although self-declaration as a rational prescriber; problematic attitudes towards prescribing	Guidance improved prescribing but still problematic attitudes; have to be improved by knowledge, guidance and communication; identification of patient factors contributing to irrational use	Survey with self-reported attitudes; limited to examined region	Quadranti et al. 2021
Impact of national programmes on prescribing behaviour	Europe	Bacterial resistance particularly addressed in Northern and Western countries vs. lack in Eastern and Southern Europe; complex relationships between factors	Identification of regions and countries that need to improve their stewardship programmes; examination of variables	Theoretical concept	Vogeler and Parth 2024
Impact of safety warning on prescription	Germany	In most cases reduced prescribing after regulatory warning, but switch to alternative often non-adherent with guideline recommendation	Regulatory restrictions have effect on consumption (often decrease); potential inappropriate choice of alternative; guidance needed	Only the drug class of fluoroquinolones considered; investigation in a single country	Georgi et al. 2021
Inconsistencies in therapeutic indications of amoxicillin	Europe	Often indication section of the pharmaceutical product information outdated, includes removed indications; arising discrepancies can cause irrational use; frequent updates necessary	Investigation and emphasising of the problem of outdated product prescriptions; need for frequent updates, monitoring and standardised procedures	Limited generalisations due to the examination of a single antibacterial drug	Opalska et al. 2025
Measurements towards improved prescribing behaviour	Europe	Minor improvements through surveillance data; missing measurements of appropriate prescribing; good health infrastructure needed	Identification of areas for improvements; investments needed to improve rational prescribing	Theoretical concept	Kern 2021

**Table 4:** Key findings from the literature review on consumption trends and surveillance aspects of antibacterial drugs. For each reference, information on the subject, region, the most important findings and their value, and the limitations is provided

Subject	Region	Take-home messages	Value of the findings	Limitations of the study	Reference
Consumption after the COVID pandemic	Italy	Strong increase of consumption; possible influence of the COVID pandemic towards irrational use; multimodal approach needed to improve prescribing	Highlights Problematic developments; provides aspects which are important to improve stewardship	Analysis of single country; short time period	Ferrara et al. 2023
Consumption and prescribing behaviour since the COVID pandemic	United Kingdom	Significant shifts in prescribing behaviour, with increasing inappropriate prescribing	Examination of stewardship programmes and rational use in health crisis; highlights need for improving and strengthening efforts and initiatives	Short time period (2019–2020), no comparison of pre- and post-pandemic situation	Elshenawy et al. 2024
Consumption of antibacterials and bacterial resistance situation	Cyprus	High awareness of bacterial resistance but lack of knowledge in the correct use of antibacterials; highest consumption in European countries; high niveau of bacterial resistance	Highlighting the importance for public understanding and a multidisciplinary approach to rational prescribing; knowledge important to ensure safe and responsible use	Potential geographical variations; more responses for the survey from single districts; potential selection bias	Michaelidou et al. 2020
Consumption trends and factors influencing antibacterial prescriptions	Germany	Increasing prescriptions until 2013, followed by slight decrease and strong COVID dip; shift in drug classes over time; strong association between decreasing costs and rising prescriptions	Overview of antibacterial drug consumption over several decades; identification of determinants, particularly costs as a driver	Observational study; cannot establish causation; limited to Germany	Bindel and Seifert 2024a
Consumption trends and impact of policy interventions	Turkey	Very high consumption in comparison with European countries; government intervention contributed to a reduced consumption and improved guideline adherence	Role of policy interventions to reduce consumption and improve prescribing behaviour	No subaggregated analyses; no consideration of antibacterial use and bacterial resistance; limited number of analysed determinants	Aksoy et al. 2021
Consumption trends	Germany	Decline between 2010 and 2018, particularly in children; potential success of stewardship programmes	Underscores the success and need for measurements to improve the appropriate prescribing and lower use of antibacterials	Major differences across German regions	Holstiege et al. 2020
Factors of bacterial resistance development in *P. aeruginosa*	Europe	Large complexity of bacterial resistance problem; North-West and South-East geographic shift; related to prescribing behaviour and national plans	Identification of government variables like corruption or gross domestic product as the most important factors; focus on national governance problems impacting resource distribution, substituted by individual guidelines and promotion of appropriate use	Data limitations; restricted number of considered determinants in analyses	Riano-Moreno et al. 2022
Impact of bacterial resistance data on prescribing	global	Physicians have to consider bacterial resistance data stronger; bacterial resistance increased because local situations were not considered in treatment	Educational interventions can change prescribing behaviour; need for frequent monitoring and access to surveillance data, user-friendly and in real-time	Reliance on considered publications, potential selection bias; not all countries represented, limiting generalisation; methodological choices	Mori et al. 2025
Impact of climate on bacterial resistance	Europe	Temperature change is not associated with bacterial resistance; main determinants are consumption, population density and governance index; improving the appropriate use and governance efficiency are most effective to prevent increasing bacterial resistance	Examination of the influence of warmer temperatures because of the climate change to bacterial resistance development	Correlation does not imply causality; gaps in national data; restricted number of pathogens; no subaggregation by smaller geographic areas	Maugeri et al. 2023
Interdependencies between antibacterial drug prescriptions	Germany	Shared patterns and significant correlation in consumption among antibacterial drugs; susceptible to external factors and mutual influence	Exploration of characteristics of prescribing patterns; better understanding possible effects of measurements	Challenges in distinguishing direct effects from common or coincidental responses to external events; focused on Germany	Bindel and Seifert 2025a

**Table 5:** Key findings from the literature review on the sociocultural and economic determinants of antibacterial prescribing. For each reference, information on the subject, region, the most important findings and their value, and the limitations are provided

Subject	Region	Take-home messages	Value of the findings	Limitations of the study	Reference
Cultural and religious influence on prescribing	Europe	High consumption in South Europe; differences stem in part from cultural and religious differences, e.g. Protestantism	Explanation of differences in the efficacy of stewardship programmes and general prescribing behaviour through cultural attitudes between North and South Europe	Limited assessment of factors which could lead to bias	Kenyon and Fatti 2020
Cultural determinants of prescribing	Greece	High antibacterial consumption, but high awareness in population; irrational prescribing partly explainable by attitudes, perceived norms and values	Role of cultural behaviour on prescribing; need of consideration when designing measurements because of risk of failure	Limited assessment of factors which could lead to bias	Papadimou et al. 2022
Impact of costs on prescriptions	Germany	Significant correlation; evidence that costs influence consumption	Highlights impact of economic factors on prescribing patterns; potential area for policy interventions	Observational study; cannot establish causality; limited to Germany	Bindel and Seifert 2024b
Problematic nomenclature	global	Misleading and inappropriate term ‘antibiotics’; contributes to confusion of patients and professionals; results in irrational prescribing and overuse; need for a renewed nomenclature	Reveals problematic communication and the hindering of appropriate treatment by outdated nomenclature; emphasises need for change to improve awareness and support knowledge	Updated nomenclature has to be implemented and established to have a positive effect on behaviour	Seifert and Schirmer 2021
Public beliefs	global	Patients have positive perception of antibacterials, strong trust in effectiveness; positive aspects more aware than problems; beliefs may contribute to high consumption	Exploration of area for policy interventions; need for targeted measures to reduce inappropriate demand for antibacterials	Online survey; selection bias; self-reported perception of patients	Jones et al. 2024

## Determinants of rational and irrational prescribing

### Attributes of rational and irrational prescribing

Rational use of drugs is defined as ‘patients receive medications appropriate to their clinical needs, in doses that meet their own individual requirements, for an adequate period of time, and at the lowest cost’ (PPRI 2023). In contrast, irrational use is defined as ‘overuse, underuse or misuse of medicines’ and ‘results in wastage of scarce resources and widespread health hazards’ (WHO 2025a). This includes ‘inappropriate use of antimicrobials, often in inadequate dosage, for non-bacterial infections; over-use of injections when oral formulations would be more appropriate; failure to prescribe in accordance with clinical guidelines; inappropriate self-medication, often of prescription-only medicines; non-adherence to dosing regimes’ (WHO 2025a).

Rational treatment requires the appropriate choice of an antibacterial drug, ideally based on pathogen identification by an antibacteriogram, considerations of bacterial resistance, drug safety profiles, dosing, duration, efficacy and patient-specific factors. It also involves avoiding unnecessary prescriptions (Littmann et al. 2020; Sweileh 2021; Ludwig et al. 2025; Seifert 2018).

Irrational use includes lack of public awareness, unrestricted drug access, insufficient medical training and guidance, pharmaceutical marketing, limited diagnostic tools, economic pressure and cultural attitudes (Machowska and Lundborg 2019; Bindel and Seifert 2025b, c). Such irrational prescribing practices are recognised in Europe and worldwide (Bindel and Seifert 2025c; ECDC 2024; Sweileh 2021; Duan et al. 2022). Inappropriate treatment can lead to treatment complications such as adverse drug effects, treatment failure, higher costs and the increase of bacterial resistance (Kilari and Oroszi 2024).

**Fig. 3:**
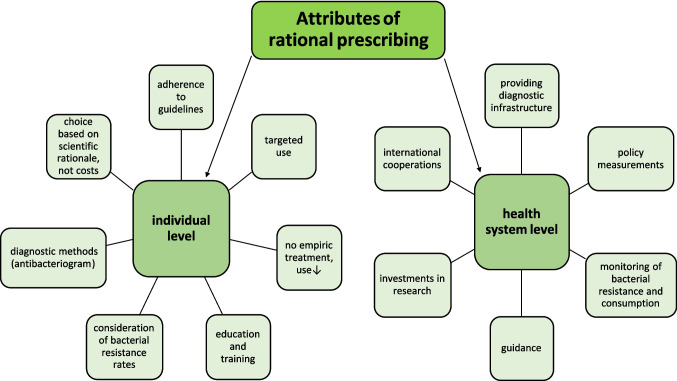
Attributes of rational prescribing at the systemic and individual level

Prescribing behaviour is shaped and influenced by multiple interrelated factors (Aricò et al. 2023; Kurotschka et al. 2020; Bindel and Seifert 2024a, 2025a). Essentially, determinants can be distinguished at systemic and individual levels. While these systems are initially independent of each other, both must be optimised to ensure rational use. Figure 3 illustrates attributes of rational prescribing.

### Systemic determinants

**Fig. 4:**
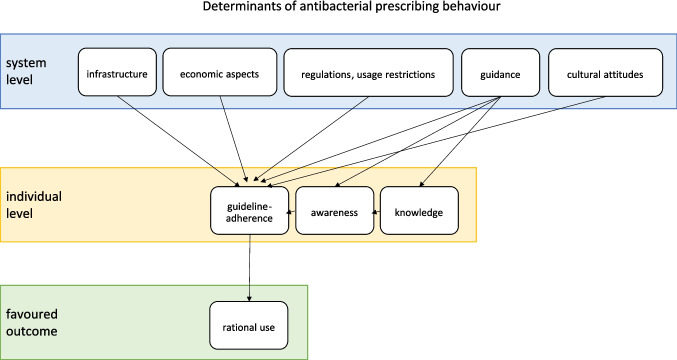
Determinants of antibacterial prescribing behaviour and possible interdependencies

Systemic factors relate to the structure of the healthcare system and consist of legislation, policies, regulations, infrastructure, resources, strategic plans and organisation (Healthcare Excellence Canada 2025). These factors are determined by the country-specific healthcare system and influence prescribing behaviour through their infrastructure, organisation and economic aspects (Kurotschka et al. 2020). They can be shaped by policy measures, guidance and cultural attitudes (Bara et al. 2022; Rose et al. 2021) (Fig. 4, Table 1). By these determinants, regional and country-specific differences in prescribing behaviour are explainable.

In general, the more resources are available and the better the access to healthcare services, the more appropriate and rational treatment can be provided (Riano-Moreno et al. 2022). For instance, without diagnostic methods such as antibacteriograms, treatment cannot identify and target specific pathogens, resulting in empirical and potentially inappropriate prescribing (Patel et al. 2024; McCleary et al. 2021). Furthermore, the presence of medical education, diagnostic stewardship tools and digital health infrastructure has a direct impact on the quality of prescribing (Shi et al. 2023; Palin et al. 2020; Holstiege et al. 2020; Mori et al. 2025; Kern 2021) (Table 3). Many studies report a lack of knowledge and practical skills among healthcare professionals (Slekovec et al. 2023; Wiese-Posselt et al. 2023; Horvat et al. 2020; Rose et al. 2021) (Table 2). In under-resourced settings, clinicians may rely more on non-specific antibacterials due to limited diagnostic support and time constraints.

Economic considerations can strongly influence prescribing patterns and contribute to irrational prescribing (Table 5). In systems where healthcare providers face budgetary pressures, such as in Germany, the cost of prescriptions is a key consideration (Bindel and Seifert 2024a, b). Similarly, in low-income countries, out-of-pocket expenses often limit patients’ access to full treatment courses, resulting in incomplete therapies (Alsan et al. 2015). Furthermore, corruption and pharmaceutical advertising also have a negative effect (Machowska and Stalsby Lundborg 2019; Rönnerstrand and Lapuente 2017).

Cultural and social attitudes play an important role. Factors such as religious beliefs and community expectations influence guideline adherence of prescribers and patient demands. For instance, in some regions like Southern Europe, patients may place pressure on providers to prescribe antibacterials (Kenyon and Fatti 2020; Jones et al. 2024; Papadimou et al. 2022; Quadranti et al. 2021) (Table 4 and 5).

Importantly, policy interventions such as stewardship programmes and usage restrictions often have a positive impact on prescribing behaviour (Table 3). Regulations and usage restrictions can reduce antibacterial consumption (Opalska et al. 2020; Georgi et al. 2021; Bindel and Seifert 2024a). Countries that have implemented structured stewardship approaches tend to report lower rates of inappropriate use (Vogeler and Parth 2024; Holstiege et al. 2020; Aksoy et al. 2021), although success varies (Papadimou et al. 2022; Kenyon and Fatti 2020). Furthermore, transparent data reporting systems and public awareness campaigns can further contribute to a more rational prescribing (Mori et al. 2025; Morrell et al. 2021; Quadranti et al. 2021; Vogeler and Parth 2024).

### Individual determinants

Individual factors are related to the decision-making process between physicians and patients. Here, too, multiple factors can influence prescribing behaviour. For professionals, combining theoretical knowledge of rational treatment with the practical ability to implement it in daily practice can be challenging. Many studies have examined knowledge gaps and feelings of unpreparedness among medical students and professionals (Slekovec et al. 2023; Wiese-Posselt et al. 2023; Horvat et al. 2020) (Table 2). This implies that awareness alone does not automatically lead to rational treatment (Michaelidous et al. 2020) in the absence of effective implementation of rational incentives.

Prescribing decisions are influenced not only by objective factors, but also by psychological factors such as expectations, fear of clinical failure and diagnostic uncertainty (Papadimou et al. 2022; Jones et al. 2024; Rose et al. 2021; Kurotschka et al. 2020) (Table 1). Cultural attitudes can also lead to unnecessary prescriptions (Jones et al. 2024; Papadimou et al. 2022) (Table 5). However, interindividual variations between prescribers are present (Cordoba et al. 2015).

The knowledge and awareness of patients varies according to education and social level (Hunter and Owen 2025; Zaniboni et al. 2021). Communication with patients is essential to find a rational treatment decision and improve treatment adherence. Effective communication with patients is essential for making rational treatment decisions and improving treatment adherence. Unfortunately, the established use of non-specific and misleading terminology makes proper communication, knowledge and therefore rational treatment difficult (Seifert and Schirmer 2021).

The existence of infrastructure does only lead to the right treatment decision if physicians are willing and able to use it, decide rationally and communicate effectively with their patients (Mori et al. 2025; Hunter and Owen 2025; Cohn et al. 2024; Kern 2021). Otherwise, rational treatment is hindered.

## Consumption and prescribing behaviour of antibacterial drugs in Europe

### Geographical variations

In Europe, there are strong geographical disparities in the prescribing behaviour (Riano-Moreno et al. 2022), concerning both consumption and the appropriate drug choice (Bindel and Seifert 2025b, c). A North-West to South-East shift is recognisable, a low consumption is paired with rational use in the North, while in the South, consumption is high and coupled with problematic prescribing behaviour (Gunnlaugsdottir et al. 2021; Aricò et al. 2023; Aksoy et al. 2021) (Tables 2 and 4). This shift is apparent in past data and current developments and is predicted to become more pronounced (Bindel and Seifert 2025b, c). There is not only a varying level, but also a differing trend with improvements in the North versus poor progress in the South. However, the COVID pandemic had a similar impact with initially decreasing but post-pandemic increasing consumption in most countries (Elshenawy et al. 2024; Ferrara et al. 2023; Ludwig et al. 2025).

Disparities are apparent not only between geographic regions, but also between neighbouring countries and within districts of a single country. Regional differences have been reported in countries such as Denmark, Sweden, Germany and Italy (Schülz et al. 2024; Bonaldo et al. 2020; Jensen et al. 2018).

Differences in consumption and prescribing behaviour are reflected in the bacterial resistance situation, with comparably low rates in the North and higher rates in the South (ECDC 2024). There is strong evidence that this is caused by the respective consumption and prescribing behaviour, with high consumption and irrational use being linked to increasing bacterial resistance (Michaelidous et al. 2020; Aricò et al. 2023; Bindel and Seifert 2025c). Other potential determinants were tested but found non-significant, such as climate or temperature (Maugeri et al. 2023). Therefore, it appears that prescribing behaviour is influenced by factors other than the natural circumstances of infections (Rose et al. 2021; Kurotschka et al. 2020; McClearly et al. 2021). The North-West to South-East shift may be the result of a combination of systematic and individual determinants regarding prescribing patterns (Kasse et al. 2024).

### Progress towards a rational treatment

Alarmingly, limited progress towards more rational prescribing was identified in both past years and in future projections. While some countries of the Northern region were able to substantially improve their use, others achieved only minimal changes or even worsened, like in Eastern Europe (Bindel and Seifert 2025b, c) (Tables 2 and 4). Indicators such as the AWaRe framework did not substantially improve and are predicted not to do so in the future. Summarised, further research confirms and generalises the problematic prescribing of antibacterial drugs in terms of quantity, quality and progress in rational treatment. Notably, no decreasing trend is observable in the consumption of antibacterials (ECDC 2024).

In recent years, the COVID pandemic caused a sharp decrease in consumption in most countries. However, this effect was just present for a short time, since consumption quickly returned to pre-pandemic levels (ECDC 2024; Ludwig et al. 2025). Worryingly, there is evidence that prescribing behaviour worsened within this time (Elshenawy et al. 2024; Ferrara et al. 2023), an effect that prevents further progress and needs to be responded to.

Most European countries are committed to achieving the targets of the ‘One Health’ approach, as set out by the European Union, the United Nations and the World Health Organization (EU 2024; Food and Agriculture Organization of the United Nations et al. 2023). By 2030, they are expected to have reduced total antibacterial consumption by 20%, implemented more rational prescribing practices and reduced the incidence of bloodstream infections caused by key pathogens (EU 2023). However, if current trends continue, the targets for consumption and rational use are unlikely to be met (Bindel and Seifert 2025b, c; ECDC 2024). Only Sweden is predicted to reach the targets for consumption and AWaRe. In contrast, for less than half of the countries, achieving the goals regarding prescribing behaviour is possible within the forecasts (Bindel and Seifert 2025b, c).

### Consequences of irrational prescribing

Antibacterial drugs are essential in modern medicine (Cook and Wright 2022). However, increasing bacterial resistance threatens their effectiveness (Salam et al. 2023; GBD Antimicrobial Resistance Collaborators 2024). Bacterial resistance and multidrug-resistant organisms are a serious public health issue with a high burden and increasing serious consequences (Antimicrobial Resistance Collaborators 2022). When antibacterial drugs become ineffective, treatment options are limited. This results in increased morbidity and mortality, prolonged illness, higher healthcare costs and the use of alternative or last-resort drugs, which further accelerates the development of bacterial resistance (Friedman et al. 2016; Salam et al. 2023; Bindel and Seifert 2025b).

The high and inappropriate use of antibacterial drugs is a well-established driver of bacterial resistance (ECDC 2024; Abejew et al. 2024). In primary care, antibacterial drugs are most frequently prescribed for respiratory infections, even though most of these are viral (McClearly et al. 2021; Ludwig et al. 2025; Gessner et al. 2023; Vellinga et al. 2023). This renders the treatment both unnecessary and ineffective, while also contributing to the development of bacterial resistance (Aricò et al. 2023; Vervloet et al 2016) (Table 1 and 4).

**Fig. 5:**
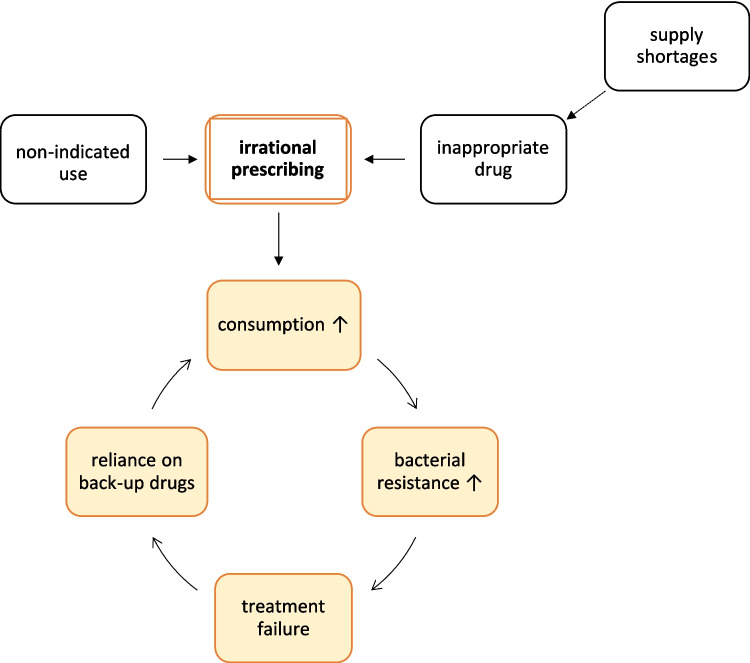
Vicious cycle (orange coloured) of irrational prescribing (cause) and bacterial resistance (effect), threatening the efficacy of antibacterial drugs. This simplified illustration is intended to provide a basic understanding of the relationship and does not capture all contributing factors

The worsening antibacterial resistance crisis is exacerbated by the fact by high resistance rates that result in the ineffectiveness of first-line antibacterial drugs, for example in Eastern Europe. This increases the dependence on ‘back-up’ drugs and accelerates the development of resistance in a vicious circle (Bindel and Seifert 2025b), as illustrated in Fig. 5.

## Future strategies to improve prescribing behaviour

### Success and requirements for regulatory measurements and stewardship programmes

Policy interventions and regulatory measurements are the foundation for an appropriate treatment and implementation of changes that support rational prescribing (Opalska et al. 2020, 2025; Georgi et al. 2021). In many countries, stewardship programmes have been effective in reducing antibacterial consumption (Aksoy et al. 2021; Holstiege et al. 2020). However, their impact varies, depending on previously discussed determinants such as healthcare infrastructure and cultural beliefs (Quadranti et al. 2021; Kern 2021; Bindel and Seifert 2025b, c; Jones et al. 2024; Elshenawy et al. 2023).

Many reports demonstrate the effectiveness of policy interventions in changing behaviour. For example, usage restrictions can lead to a reduction in the use of a particular antibacterial drug or drug class (Georgi et al. 2021; Bindel and Seifert 2024a). However, regulatory restrictions alone are insufficient; guidance must also be provided to ensure that the induced change leads to improved prescribing behaviour (Georgi et al. 2021). This can include training, guidelines or stewardship programmes to raise awareness among healthcare workers and the public (Table 3).

Many European countries have implemented strategies to improve antibacterial use (ECDC 2023). For example, countries that have strengthened their efforts to reduce antibacterial consumption have been able to successfully influence consumption trends (Oberjé et al. 2017), exhibiting better prescribing behaviour than other, neighbouring countries (Bindel and Seifert 2025b, c). Conversely, countries with underdeveloped strategies exhibit higher consumption and more irrational prescribing behaviour than their neighbours (Wojkowska-Mach et al. 2018) (Table 1 and 4).

As countries are interconnected, it is important to follow a similar strategy internationally (Opalska et al. 2020, 2025; Cohn et al. 2024; Patel et al. 2024; Elshenawy et al. 2023). In the past years, several programmes and targets were implemented, such as the ‘One Health’ approach (WHO 2025b; EU 2024). Tools like the AWaRe framework of the WHO provide a universal guide for rational use (Mudenda et al. 2023). It is important to harmonise and update recommendations and regulations (Opalska et al. 2020, 2025; Bindel and Seifert 2025b). Many stewardship models have been introduced (Morrell et al. 2021; Kern 2021; Cohn et al. 2024; Patel et al. 2024) (Table 3), but action is needed to implement these concepts in the real world.

### The need for a renewed nomenclature

**Fig. 6:**
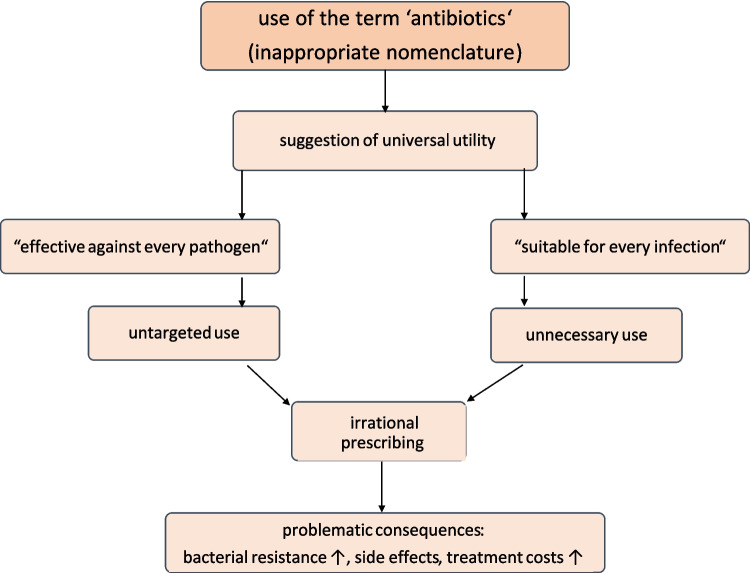
Problematic developments arising from the use of the term ‘antibiotics’ and inappropriate nomenclature in general

Some years ago, the nomenclature of terms associated with antibacterial drugs began to be rethought. Established terms such as ‘antibiotic’ and ‘broad-spectrum’ are often problematic because they are imprecise and misleading. This makes communication and understanding difficult, potentially leading to irrational prescribing (Fig. 6). Therefore, specific terms such as ‘antibacterial’ and ‘antibacteriogram’ should be used to avoid confusion or misuse (Seifert and Schirmer 2021).

**Fig. 7:**
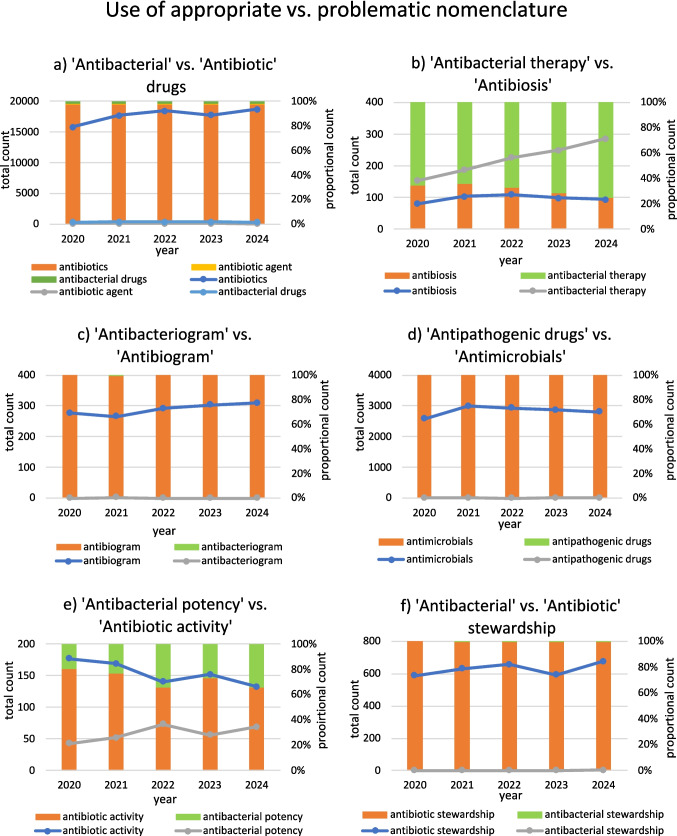
Comparison of the use of appropriate versus problematic nomenclature. The respective terms are compared in terms of their absolute count and proportion. Depicted are the years 2020–2024. Appropriate terms are highlighted in green and problematic terms are highlighted in orange or yellow. Compared groups include ‘antibacterial drugs’ versus ‘antibiotics’ and ‘antibiotic agent’ (**a**), ‘antibacterial therapy’ versus ‘antibiosis’ (**b**), ‘antibacteriogram’ versus ‘antibiogram’ (**c**), ‘antipathogenic drugs’ versus ‘antimicrobials’ (**d**), ‘antibacterial potency’ versus ‘antibiotic activity’ (**e**), and ‘antibacterial stewardship’ versus ‘antibiotic stewardship’ (**f**)

To investigate the progress towards a more appropriate nomenclature, the analysis conducted by Seifert and Schirmer (2021) was updated for the subsequent four years. The total number of citable items and the normalised count were analysed, and comparison was conducted between the use of the respective problematic and appropriate terms. A PubMed search revealed an increasing use of most terms, except for some that are used very rarely, such as ‘antibacteriogram’ or ‘antibiotic activity’. However, when comparing the use of the problematic and appropriate terms, the problematic terms are used much more frequently. An exception is ‘antibiosis’ versus ‘antibacterial therapy’, where the appropriate term was used three times more often in 2024. The ratio for all other pairs is between 1.9:1 and 1618:1 (problematic to appropriate). A detailed analysis of the results is provided in Table 6, Fig. 7 and in the supplement.

**Table 6:** Update of the findings from Seifert and Schirmer (2021) regarding the use of appropriate versus problematic nomenclature (update May 12 2025). Progress is compared for the entire available time period since 1965 and for recent years since 2020, with regard to the normalised count (i.e. publications using the respective term divided by the total citable items in PubMed). Problematic terms and ratios are highlighted in orange, while rational nomenclature and progress are highlighted in green

Term	Mean of normalized count 1965-2020	Mean of normalized count 2020-2024	Trend in the whole time period (normalized count)	Recognizable change since 2020 (normalized count)	Ratio problematic vs. appropriate nomenclature in 2020 (normalized count)	Ratio problematic vs. appropriate nomenclature in 2024 (normalized count)	Progress regarding the ratio of appropriate vs. problematic nomenclature	Comment
Antibiotics	5.481	10.244	↑	No	52.7:1	55.6:1	↓	Regress towards problematic use and dominance of inappropriate term
Antibiotic agent	0.041	0.079	↑	Stabilization in use	52.7:1	55.6:1		
Antibacterial drugs	0.086	0.207	↑	Stabilization at a low level	1:52.7	1:55.2		
Antibiosis	0.023	0.056	↑	Stabilization in use	1:1.9	1:3.06	↓	Regress towards problematic use, appropriate term yet more often used
Antibacterial therapy	0.079	0.128	↑	Increasing use	1.9:1	3.06:1		
Antibiogram	0.095	0.168	↑	Increasing use	-	-	→	Established use of problematic term
Antibacteriogram	0	0	→	Not used	-	-		
Antimicrobials	0.483	1.643	↑	Increasing use	796:1	1618:1	↓	Established use of problematic term
Antipathogenic drugs	0	0.001	↑	Increasing but marginal use	1:796	1:1618		
Antibiotic activity	0.076	0.089	→	Stabilizing	4.2:1	1.9:1	↑	Progress towards rational nomenclature
Antibacterial potency	0.012	0.034	↑	Increasing use	1:4.2	1:1.9		
Antibiotic stewardship	0.036	0.365	↑	Increasing use	-	195.5:1	↑	Continued and increasing use of problematic term, low progress in regard to improved nomenclature
Antibacterial stewardship	0	0.001	↑	Increasing but marginal use	-	1:195.5

When assessing progress by comparing the ratio of the normalised count between 2020 and 2024, an increasing preference for the inappropriate term is evident for most pairs, indicating a worsening trend. The exceptions are ‘antibacterial/antibiotic stewardship’ and ‘antibiogram/antibacteriogram’, which show a marginal improvement towards the appropriate terminology. In terms of absolute citation counts, both the appropriate and inappropriate terms increased. However, the problematic terms remained dominant and increased at a faster rate.

These findings imply only marginal improvements in the adoption of a renewed nomenclature. Despite growing awareness and strengthened efforts in stewardship, there is a lack of focus on terminology and communication. Therefore, this reform must be introduced into clinical education, scientific journal policies, guidelines and official communication to establish a precise nomenclature and communication.

### Proposals for action

**Fig. 8:**
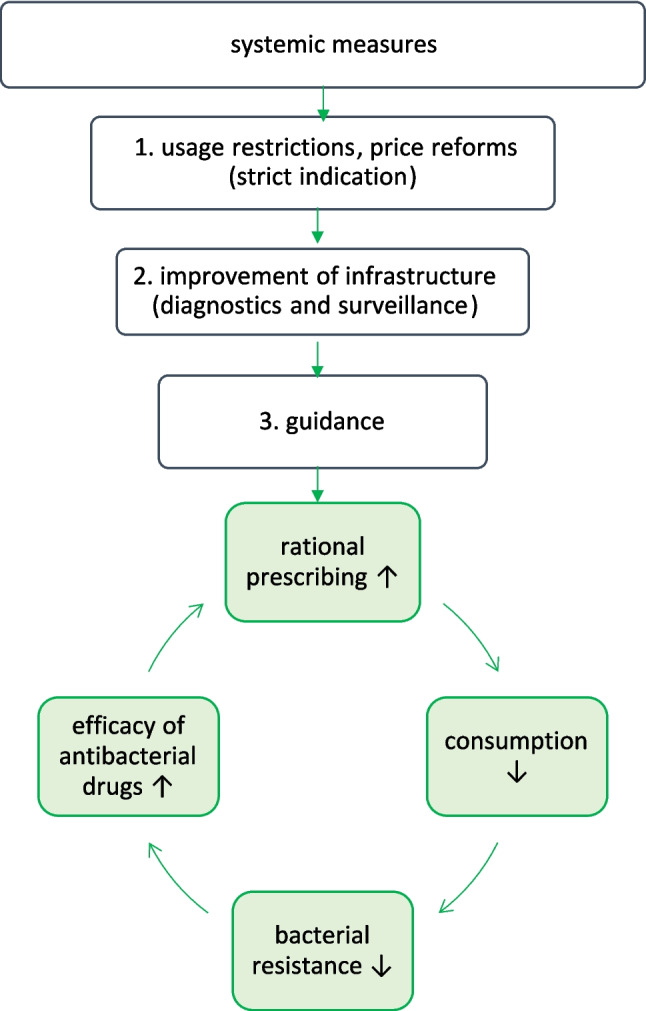
Action plan to improve rational prescribing and solve the ‘bacterial resistance crisis’. This step-by-step plan shows which initial policy interventions are needed. This simplified overview is intended to highlight key steps and does not encompass all possible measures or complexities

In general, it is very important to focus on policy measurements for targeted factors. Our findings suggest that systemic changes are required to improve individual behaviour. At the systemic level, establishing a comprehensive and effective infrastructure is essential. This includes resources such as diagnostic methods, accessibility and reliability of healthcare and the availability of antibacterial drugs. The lack of these foundations leads to irrational use, as seen in Eastern countries with a lower expenditure on healthcare (Eurostat 2024). Supply shortages that lead to the unavailability of first-line drugs must be prevented (Bindel and Seifert 2025d). Furthermore, policy measurements must be adjusted to restrict the use of antibacterial drugs and support the rational use. For example, economic factors are a relevant determinant that can be directly addressed (Bindel and Seifert 2024a). Consequently, substantially increasing the price of drugs that require careful prescription can reduce the consumption of the respective drug (Bokhari et al. 2024). In general, it is important to reduce the reliance on economic considerations in treatment decisions. However, regulatory restrictions must be accompanied by programmes and guidance, including up-to-date guidelines, improved training of professionals and public information campaigns (Patel et al. 2024). Otherwise, prescribing behaviour is shifting to other antibacterial drugs but not necessarily improving (Georgi et al. 2021). Figure 8 introduces initial steps for improving prescribing behaviour through systemic adjustments.

In combination with system-wide measures, there is a need to improve individual prescribing behaviour towards more rational practices (Bindel and Seifert 2024b, c), for example by the identification of high-volume prescribers (Graber et al. 2024). It is important to set strict indications for antibacterial drugs to avoid unnecessary treatment in viral infections. In general, antibacterial drugs must be used as sparingly as possible. A targeted use with an effective and specific antibacterial drug is important. Therefore, the causing pathogen must be identified by an antibacteriogram. Continuous updating on current guideline recommendations and the local bacterial resistance situation is necessary. DDD costs must be ignored, since the choice of a drug must rely on scientific evidence and not economic considerations (Bindel and Seifert 2025c). Moreover, indirect costs resulting from treatment complications, adverse drug effects and increasing bacterial resistance are much higher (Antimicrobial Resistance Collaborators 2022; O’Neill 2014) than the differing costs in antibacterial drugs or by the establishment of rational practices.

Without a functioning health system, there is no foundation for the individual to establish rational use. Nevertheless, even if a good foundation is available, it must be used for each single treatment situation. Investment in research is required to develop new drugs and therapeutic approaches (Dance 2024), as well as to monitor ongoing developments (EU 2023; Fuhrmeister and Jones 2019).

The goal of all measures is to establish rational prescribing in order to reduce bacterial resistance rates and preserve the effectiveness of available treatment options. Otherwise, the burden of multidrug-resistant infection will increase substantially, resulting in millions of deaths related to bacterial resistance, and substantial costs will arise (Antimicrobial Resistance Collaborators 2022). It is therefore very important to reduce the consumption of antibacterial drugs to limit the increase of bacterial resistance. This must be supported by a very restrictive use of antibacterial drugs, strict adherence to guideline recommendations (Biniek et al. 2024; Oliveira et al. 2020) and awareness of the current bacterial resistance situation (Mori et al. 2025).

## Limitations

Several limitations must be acknowledged. Due to the restrictions on keywords, time period and geographic region, it is possible that key literature was overlooked in the PubMed search. Furthermore, the considered literature had a heterogeneous design and methodology. Assessing prescribing behaviour and key determinants is complex and influenced by analyses that cannot be fully objectified. Furthermore, correlation does not imply causation, restricting the conclusions that can be drawn from statistical findings. In general, it is possible that determinants forming or shaping prescribing behaviour have been overlooked. The proposals for action provided are basic recommendations that must be implemented as specific measures.

## Conclusions

Prescribing behaviour follows complex patterns, being determined and influenced by multiple interconnected factors (Bindel and Seifert 2024a; Riano-Moreno et al. 2022; Vogeler and Parth 2024; Bara et al. 2022) (Fig. 3, Tables 1, 2, 3, 4 and 5). It is a challenge to identify the main drivers and to design targeted measures, as the determining cause is often not straightforward. Beyond recognising the driver, statistical significance or correlations alone do not imply causality. Once the key drivers have been identified, targeted and effective measures must be implemented to successfully initiate change. In the past, such measures succeeded in changing behaviour, but with varying extents (Quadranti et al. 2021;Vogeler and Parth 2024; Bara et al. 2022).

Prescribing behaviour is often considered from an individual perspective (Murshid and Mohaidin 2017), with studies focusing on the drivers of personal decisions and measures aimed at changing individual behaviour (Rose et al. 2021; Kurotschka et al. 2020). It is often overseen that systemic determinants are the foundation of individual prescribing behaviour (Fig. 4). Circumstances therefore have a strong influence on individual treatment. Notably, the findings indicate a strong influence of systemic determinants on prescribing behaviour, being often underestimated. Strong drivers are health system–related factors like infrastructure, resources and financial organisation, policy interventions like usage restrictions and guidance, or cultural attitudes (Bindel and Seifert 2024a, b, 2025a, b; Kenyon and Fatti 2020; Maugeri et al. 2023; Riano-Moreno et al. 2022). Prescribing behaviour is largely determined by systemic rather than individual factors, as suggested by the North–South shift in Europe (Kenyon and Fatti 2020; Bindel and Seifert 2025b, c) and by similar patterns observed in other unrelated drug classes (Bindel and Seifert 2025e).

Therefore, the primary focus should be on systemic measures, supplemented by efforts to improve individual behaviour (Opalska et al. 2020, 2025) (Fig. 8). One advantage of this strategy is that structural issues, such as diagnostic infrastructure, access to first-line drugs, clinical guidelines and pricing or usage policies, can often be addressed more directly than individual behaviours, which are more diffuse and variable. Although awareness among healthcare professionals and the public appears to be increasing (Quadranti et al. 2021; Horvat et al. 2020), this awareness often fails to translate into practice due to systemic issues such as drug shortages, a lack of diagnostic capacity or lack of regional bacterial resistance data (Slekovec et al. 2023; Wiese-Posselt et al. 2023; Papadimou et al. 2022; Bindel and Seifert 2025d; Turner et al. 2023; Bertagnolio et al. 2023). This suggests that systemic issues are a key factor in achieving rational treatment.

Improving antibacterial drug prescribing is particularly important since it directly affects public health, not just individual treatment. Increasing bacterial resistance places a huge burden on the health care system, consuming time and financial resources, and poses a serious threat to patient health (Antimicrobial Resistance Collaborators 2022). Despite initiatives to stop increases, bacterial resistance has risen in recent years, reflecting the poor progress reported (ECDC 2024; Bindel and Seifert 2025b, c). Prescribing behaviour appears to be directly linked to bacterial resistance (Fig. 5), as evidenced by lower bacterial resistance and more rational prescribing in Northern regions versus the problematic situation in the South. Even the availability of a new antibacterial drug will only provide short-term relief if irrational prescribing practices and determinants persist (Cormican and Vellinga 2012). Addressing the ‘bacterial resistance crisis’ is only possible when a rational prescribing behaviour is implemented, characterised by low consumption and appropriate drug selection. Otherwise, the bacterial resistance situation will continue to worsen, with serious consequences.

## Supplementary Information

Below is the link to the electronic supplementary material.

Supplementary file (XLSX 81.5 KB)

## Data Availability

All source data for this study are available upon reasonable request from the authors.

## Abbreviations

AWaRe: Classification of antibacterial substances to support rational use and monitoring antibacterial consumption (divided into groups ‘Access’, ‘Watch’, ‘Reserve’) (WHO ( 2023)
DDD: Defined daily dose
DID: Defined daily dose prescriptions per 1000 inhabitants per day
ECDC: European Centre for Disease Prevention and Control (public health agency of the EU)
EU: European Union
PubMed: Database for biomedical and life sciences literature, maintained by the U.S. National Center for Biotechnology Information (https://pubmed.ncbi.nlm.nih.gov/)
UN: United Nations
WHO: World Health Organization
